# A Robust High-Accuracy Ultrasound Indoor Positioning System Based on a Wireless Sensor Network

**DOI:** 10.3390/s17112554

**Published:** 2017-11-06

**Authors:** Jun Qi, Guo-Ping Liu

**Affiliations:** 1 School of Astronautics, Harbin Institute of Technology, Harbin 150001, China; gpliu@hit.edu.cn or guoping.liu@southwales.ac.uk; 2 School of Engineering, University of South Wales, CF37 1DL Pontypridd, Wales, UK

**Keywords:** ultrasonic indoor positioning system, wireless sensor network, time-of-flight for ultrasonic signal, least squares method

## Abstract

This paper describes the development and implementation of a robust high-accuracy ultrasonic indoor positioning system (UIPS). The UIPS consists of several wireless ultrasonic beacons in the indoor environment. Each of them has a fixed and known position coordinate and can collect all the transmissions from the target node or emit ultrasonic signals. Every wireless sensor network (WSN) node has two communication modules: one is WiFi, that transmits the data to the server, and the other is the radio frequency (RF) module, which is only used for time synchronization between different nodes, with accuracy up to 1 μs. The distance between the beacon and the target node is calculated by measuring the time-of-flight (TOF) for the ultrasonic signal, and then the position of the target is computed by some distances and the coordinate of the beacons. TOF estimation is the most important technique in the UIPS. A new time domain method to extract the envelope of the ultrasonic signals is presented in order to estimate the TOF. This method, with the envelope detection filter, estimates the value with the sampled values on both sides based on the least squares method (LSM). The simulation results show that the method can achieve envelope detection with a good filtering effect by means of the LSM. The highest precision and variance can reach 0.61 mm and 0.23 mm, respectively, in pseudo-range measurements with UIPS. A maximum location error of 10.2 mm is achieved in the positioning experiments for a moving robot, when UIPS works on the line-of-sight (LOS) signal.

## 1. Introduction

Satellite signaling systems are universally well known. One example is the global positioning system (GPS), which has a high positioning accuracy at 0.15 m [1]. It is very convenient to use satellites to obtain locations. Unfortunately, satellite positioning signals normally cannot be received indoors due to obstructions from buildings, and people have to do most of their work (an estimated 87 percent overall) indoors [2].

Because of the reasons outlined above, indoor positioning systems (IPSs) have gained increasing attention from researchers across the globe in the last few decades, as IPSs are widely used in health-care as well as in home and building automation [3,4,5,6]. There are already many local positioning systems based on different technologies, and they each have their own advantages and weaknesses. Systems based on infrared beams make use of the scanning sweep method and are very inexpensive for users, but only have accuracy up to 57 cm [7]. Xiaohan Liu et al. [8] developed an IPS using a visible light communication platform, which has an accuracy of 10 cm, but it requires expensive infrastructure. The ultra-wide-band (UWB) is a great option for IPS and has a strong anti-interference capability, but it needs more infrastructure and is expensive for users [9]. IPS based on computer vision [10] has the highest precision of the all technology proposals, but it is too expensive and lacks wide applications. The other local positioning solutions consist of radio frequency identification (RFID) [11], WiFi [12], and ZigBee [13], and they have a low positioning accuracy.

Compared to these above technical solutions, a positioning system based on the ultrasonic signal and a wireless sensor network (WSN) has many advantages, including slow propagation speed (about 340 m/s), so that the hardware for the received ultrasonic signals can be simple, obtaining cost savings. The time-of-flight (TOF) of the signal from a transmitter device to a receiver is used to calculate the distance in the UWB and ultrasonic solutions, but a lower time synchronization accuracy between different nodes can be tolerated for the ultrasound’s speed, compared to the speed of electromagnetic wave propagation in the UWB system. The ultrasound signal is weakly influenced by the surroundings and has a negligible penetration of walls, and this can enhance its practical value in the proposed ultrasonic indoor positioning system. Besides, ultrasonic transducers can be integrated easily into the mobile phone system [14]. This is almost equivalent to adding a new human organ, and the location of the person is conveniently completed by it.

There are already many methods of ultrasonic signal processing for distance measurement, including non-destructive testing [15,16]. The envelope signal extracted by these methods has some harmonics that cannot be ignored, and this will affect the measurement accuracy if they are used in ultrasonic indoor positioning systems (UIPSs). The Cricket location system [17] detects the arrival of the ultrasound with a threshold value. It is simple, but has low precision due to noise interference. The random signal processing method is used to estimate ultrasonic TOF in some works in the literature [18,19], considering environmental noise, but these algorithms are too complex for UIPSs based on WSNs. The ultrasonic signals are encoded into various digital codes, such as binary frequency shift-keyed and code division multiple access codes [20,21,22,23]. These methods can indeed improve the stability of system, but are difficult to decode because of ultrasonic vibration inertia. The acoustical signals are modulated into four sets of orthogonal chirps with multiple access to solve signal interference in [24], but the chirp signals in conjunction with cross correlation are very complex, and the time resolution of the system for position will be reduced. The frequency-hopping spread spectrum (FHSS) has been introduced into the field of IPS to solve the multiple-access problem [25,26], but the ultrasonic transducer has a narrow frequency bandwidth, which will increase the difficulty and cost of realizing FHSS. Quadrature sampling is used to detect the ultrasonic envelope with a low frequency [27], which is appropriate for the WSN system and can save power, but it has low robustness. Power is not a problem. Considering that the positioning system works indoors, a new envelope detection method (EDM) is proposed in this paper. The EDM can obtain a high accuracy and robustness in UIPSs.

The paper is organized as follows. In Section 2, the UIPS architecture is described. A detailed account of the principle of the EDM is given in this section. Section 3 describes the algorithm to measure the TOF of the ultrasonic signal and the location of the target. The implementation of the UIPS is shown in Section 4, including the hardware and software. Section 5 gives the experiment results for the EDM in the simulation and practical applications. Finally, the main conclusions of the whole work are summarized in Section 6.

## 2. Global Description of the UIPS

### 2.1. UIPS Architecture

**Figure 1:**
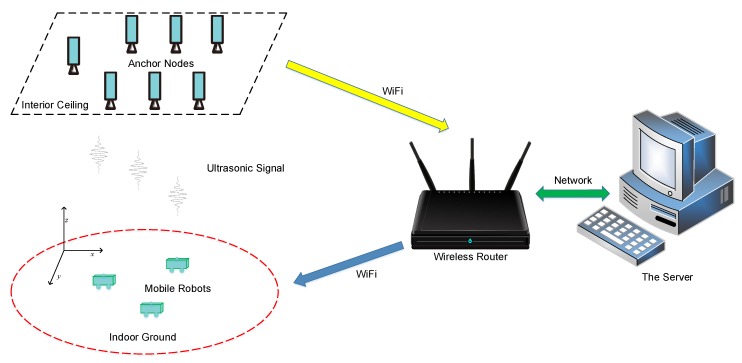
The architecture of the ultrasonic indoor positioning system (UIPS).

The UIPS has many WSN anchor nodes that are placed at known positions of the indoor space, as shown in Figure 1. The anchor nodes are on the interior ceiling, and there is a WSN node on the mobile robot system, which is the positioning target. The distance values from the target node to every anchor node and their address and time information will be transmitted to the server via wireless WiFi, when all distance measurements are finished. The coordinates for the mobile robot can be calculated by the server when the distance values to all anchors in the same moment are received. The server can forward these coordinates on to users (robots here).

**Figure 2:**
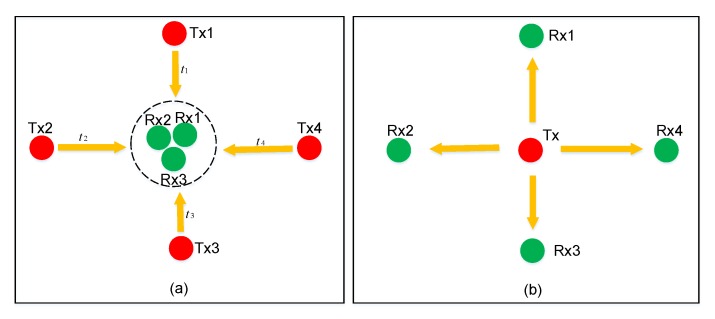
The two work modes for the ultrasonic signal: (**a**) and (**b**), Tx: transmitter, Rx: receiver.

There can be two work modes to transmit the ultrasonic signal: the first one is from the anchor nodes to the robot, shown in Figure 2a, and the second is vice versa, as shown in Figure 2b.

We assume that the number of the anchors is $N_{1}$ ($N_{1}>3$), and the number of robots is $N_{2}$ ($N_{2}>0$) in a coverage area. Every anchor will send the ultrasonic signal separately at a different time, presented as $t_{1}$, $t_{2}$, ⋯ in Figure 2a. Thus, the signal interference between different anchor nodes is avoided when the time synchronization signal (TSS) arrives in the first work mode. There is some time drift between different anchors, for which there must be a compensation algorithm, but it is very complex, and the accuracy is not high. The advantage with this mode is that it needs only one TSS for positions of all robots, and saves power. If just one anchor transmits the signal in a TSS in order to void the compensation for time drift, there needs to be $N_{1}$ TSSs for all robots, but the position will have large errors in both methods above when robots are moving. Because the measurements for the distances to all anchors are finished at different times, the position coordinates can only be calculated roughly through these distance values.

In the second work mode, the target nodes transmit the ultrasonic signal to the anchors, selected by the UIPS in this paper. Only one robot sends the signal that will be received by all anchors at the same time when the TSS for it arrives, and then the distances to all anchors are measured. The ultrasonic signal pulse is just 1 ms, which will be described later in this article. There is a reasonable assumption that the position for the moving robot will not change in so short a time, so this mode can be appropriate for mobile robot localization, although it needs more TSSs ($N_{r}$) for all robots. The information from the time synchronization node contains a robot address. The robot with this address will send the ultrasonic signal, and the remaining ones will not. The anchors distinguish the ultrasonic signals from each other with the address. The time synchronization node will cyclically transmit TSS to every robot in turn, so the resolution for UIPS will reduce when $N_{r}$ grows larger.

### 2.2. Ultrasonic Envelope Detection

Ultrasound is a vibration wave, for which the amplitude will increase when it arrives at the peak. There will be a reducing process while the signal drops to the minimum amplitude value from the peak value. If the driving signal for the ultrasound is cut off immediately when the peak arrives, the signal will have a unique wave peak. The signal for this means can be described by Equation (1) [28].

(1) $$x\left(t\right)=A\left(t\right)\cdot \sin(2\pi f_{c}t+\theta \left(t\right))$$

where the parameters A(t), $f_{c}$, and θ(t) are the signal amplitude, frequency, and phase, respectively. A(t) is also the envelope signal and is given by:

(2) $$A\left(t\right)=\beta e^{-\alpha {\left(t-\tau \right)}^{2}}$$

where β is the amplitude parameter, and α is the bandwidth factor. We can see that A(t) will obtain its maximum value when t=τ from the equation.

**Figure 3:**
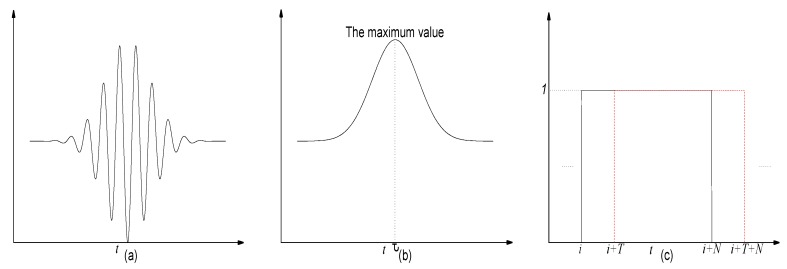
(**a**) Ultrasonic signal; (**b**) Envelope; and (**c**) Rectangular window.

The original ultrasonic signal x(t) from the received transducer is shown in Figure 3a, and the envelope A(t) is presented in Figure 3b. The ultrasonic envelope detection aims to extract A(t) from x(t). We consider that the time when the ultrasonic envelope arrives at its peak is the arrival time of the ultrasonic signal. That means the TOF for the ultrasonic envelope is τ in Equation (2), if t=0 is the start time for transmission of the signal. A rectangular window function w(t) with a length of *N* is defined as Equation (3). It will become w(t−T), w(t−2T), and w(t−3T), ⋯, when the function is moving with a step of *T*, as shown in Figure 3c. *T* is the sampling period for the ultrasound signal. x(t) of the length of N·T is selected in every window, when x(t) multiplies the window function.

(3) $$w\left(t\right)=\left\{\begin{array}{cc} 1 & 0\;<\;t\;\leq \;NT\\ 0 & t\;\mathrm{is}\;\mathrm{others}. \end{array}\right.$$

The digitalized values x(n) are provided by the analog to digital converter (ADC) from x(t) with a sampling rate of $f_{s}$ (1/T), given by:

(4) $$\begin{array}{cc} x(n) & =A\left(n\right)\cdot sin(w_{c}nT+\theta \left(n\right))\\ & =A\left(n\right)\cdot sin\left(w_{c}nT\right)cos\left(\theta \left(n\right)\right)+A\left(n\right)\cdot cos\left(w_{c}nT\right)sin\left(\theta \left(n\right)\right)\\ & =\left[\begin{array}{c} sin\left(w_{c}nT\right)cos\left(w_{c}nT\right) \end{array}\right]\left[\begin{array}{c} A(n)cos(\theta (n))\\ A(n)sin(\theta (n)) \end{array}\right] \end{array}$$

where A(n) and θ(n) stand for the amplitude sampling value of the envelope and the phase angle at nT time, respectively, and $w_{c}=2\pi f_{c}$.

If the window function w(t) is discretized into w(n) with the same frequency $f_{s}$, and we can get Equation (5) in the window *i*,

(5) $$\begin{array}{c} \tilde{x}\left(i+j\right)=x\left(i+j\right)\cdot w\left(n-i+j\right) \end{array}$$

where j=1,2,⋯,N and i=0,1,2,⋯. Because the value of N·T is small, at just 0.05 ms in this paper (where N=50 and T=1μs), ultrasonic signal is a mechanical vibration, with the characteristic of inertia. Its amplitude and phase have little change in such a short period of time. We can assume that the envelope value A(i+j) and the phase θ(i+j) are constant for all *j* values in every window *i*, presented as $\tilde{A}\left(i\right)$ and $\tilde{\theta }\left(i\right)$, respectively, and then we can obtain Equation (6) from Equations (4) and (5), where $\tilde{x}\left(i+1\right)$, $\tilde{x}\left(i+2\right)$, $\cdots ,\tilde{x}\left(i+N\right)$ can be obtained from ADC sampling.

(6) $$\begin{array}{ccc} \tilde{x}\left(i+1\right) & = & sin\left(w_{c}\left(i+1\right)T\right)\cdot \tilde{A}\left(i\right)cos\left(\tilde{\theta }\left(i\right)\right)+cos\left(w_{c}\left(i+1\right)T\right)\cdot \tilde{A}\left(i\right)sin\left(\tilde{\theta }\left(i\right)\right)\\ \tilde{x}\left(i+2\right) & = & sin\left(w_{c}\left(i+2\right)T\right)\cdot \tilde{A}\left(i\right)cos\left(\tilde{\theta }\left(i\right)\right)+cos\left(w_{c}\left(i+2\right)T\right)\cdot \tilde{A}\left(i\right)sin\left(\tilde{\theta }\left(i\right)\right)\\ \tilde{x}\left(i+3\right) & = & sin\left(w_{c}\left(i+3\right)T\right)\cdot \tilde{A}\left(i\right)cos\left(\tilde{\theta }\left(i\right)\right)+cos\left(w_{c}\left(i+3\right)T\right)\cdot \tilde{A}\left(i\right)sin\left(\tilde{\theta }\left(i\right)\right)\\ \;\;\;\;\cdots \;\;\; & = & \;\;\;\;\;\;\;\;\;\;\;\;\;\;\;\;\;\;\;\;\;\cdots \;\;\;\;\;\;\;\;\;\;\;\;\;\;\;\;\;\;\;\;\;+\;\;\;\;\;\;\;\;\;\;\;\;\;\;\;\;\;\cdots \\ \tilde{x}\left(i+N\right) & = & sin\left(w_{c}\left(i+N\right)T\right)\cdot \tilde{A}\left(i\right)cos\left(\tilde{\theta }\left(i\right)\right)+cos\left(w_{c}\left(i+N\right)T\right)\cdot \tilde{A}\left(i\right)sin\left(\tilde{\theta }\left(i\right)\right) \end{array}$$

Equation (6) is written in the form of a matrix:

(7) $$X_{i}=\Psi_{i}\cdot C_{i}$$

where the transposes of $X_{i}$, $C_{i}$ and $\Psi_{i}$ are given by:

(8) $$X_{i}^{T}=\left[\begin{array}{ccccc} \tilde{x}\left(i+1\right) & \tilde{x}\left(i+2\right) & \tilde{x}\left(i+3\right) & \ldots & \tilde{x}\left(i+N\right) \end{array}\right]$$

(9) $$C_{i}^{T}=\left[\begin{array}{cc} \tilde{A}\left(i\right)cos\left(\tilde{\theta }\left(i\right)\right) & \tilde{A}\left(i\right)sin\left(\tilde{\theta }\left(i\right)\right) \end{array}\right]$$

(10) $$\Psi_{i}^{T}=\left[\begin{array}{cccc} sin(w_{c}\left(i+1\right)T) & sin(w_{c}\left(i+2\right)T) & \ldots & sin(w_{c}\left(i+N\right)T)\\ cos(w_{c}\left(i+1\right)T) & cos(w_{c}\left(i+2\right)T) & \ldots & cos(w_{c}\left(i+N\right)T) \end{array}\right]$$

Now, the problem is transformed into a method to estimate $C_{i}$ of Equation (7). The estimation of $C_{i}$ is ${\hat{C}}_{i}$ (assumed ${\hat{C}}_{i}=\left[\begin{array}{cc} {\hat{c}}_{i1} & {\hat{c}}_{i2} \end{array}\right]$), and it can be determined using the least squares method for minimizing the following error function between $X_{i}$ and ${\tilde{X}}_{i}$ (${\tilde{X}}_{i}=\Psi_{i}\hat{C_{i}}$):

(11) $$J\left(\hat{C_{i}}\right)={\left(X_{i}-\Psi_{i}\hat{C_{i}}\right)}^{T}\left(X_{i}-\Psi_{i}\hat{C_{i}}\right)$$

We can obtain Equation (12) in order to minimize $J(\hat{C_{i}})$:

(12) $${\left.\frac{\partial J}{\partial C_{i}}\right|}_{C_{i}=\hat{C_{i}}}=-2\Psi_{i}^{T}\left(X_{i}-\Psi_{i}\hat{C_{i}}\right)=0$$

and then, $\hat{C_{i}}$ is given by:

(13) $$\hat{C_{i}}={\left(\Psi_{i}^{T}\Psi_{i}\right)}^{-1}\Psi_{i}^{T}X_{i}$$

we can get $\hat{A}\left(i\right)$ and $\hat{\theta }\left(i\right)$, which are the estimations of $\tilde{A}\left(i\right)$ and $\tilde{\theta }\left(i\right)$, respectively, from Equations (9) and (13):

(14) $$\hat{A}\left(i\right)=\sqrt{{\hat{c}}_{i1}^{2}+{\hat{c}}_{i2}^{2}}$$

(15) $$\hat{\theta }\left(i\right)=arctan\left(\frac{{\hat{c}}_{i2}}{{\hat{c}}_{i1}}\right)$$

## 3. Implementation of Positioning

The received nodes must know the time at which the transmitter node starts to generate the ultrasonic signal. Hence, there must be a time synchronization in these nodes, which is presented in the first part of this section, followed by TOF estimation and positioning.

### 3.1. Time Synchronization

**Figure 4:**
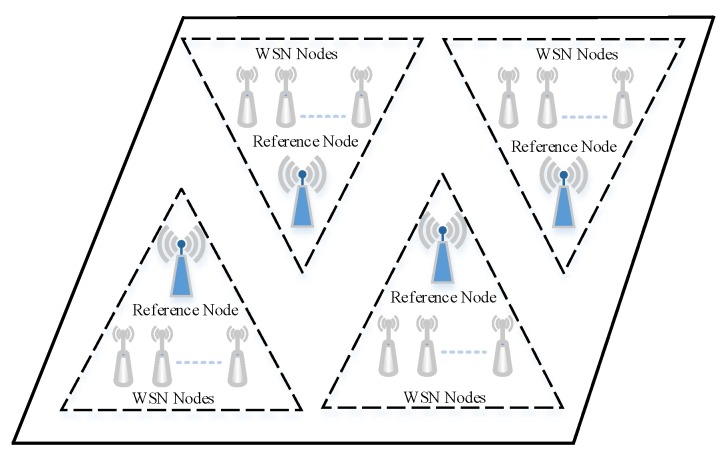
The architecture of time synchronization among different nodes.

The time protocol of the UIPS is based on reference broadcast synchronization (RBS) [29], which works in the data link layer. The architecture of synchronization for the UIPS is shown in Figure 4.

The whole space is divided into several subspaces, and there is a reference node in every subspace. All anchor nodes of the subspace have a common ultrasonic signal coverage region, and the RBS node will be asked to broadcast the synchronization signal periodically to the anchor nodes and the target nodes by the server, when there are some positioning nodes that walk into the region. The transmitters will start to work, and the received nodes will begin sampling the signal as the synchronization broadcast is arriving.

**Figure 5:**
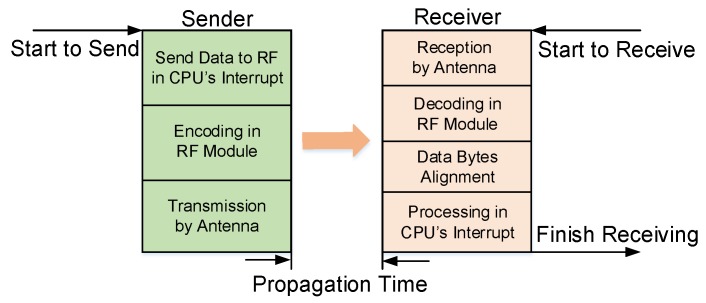
The process of the transmission for the time synchronization signal (TSS). RF: radio frequency.

The process of the transmission for TSS is shown in Figure 5. The central processing unit (CPU) of the reference node will start a timer count when it receives instructions from the server. The timer will generate an interrupt signal and count from zero again while the counter arrives at the TSS period, and then the CPU program will access the interrupt handing, where the synchronization frames are transmitted to the RF module. The frames will be encoded in the RF module and then transmitted to the receivers by the antenna. Receiving and decoding will be completed by the receivers with a reversed order of the sender when the TSS arrives at the receiving systems. Furthermore, the alignment for the received frames is finished and then an interrupt signal is generated for the CPU.

The velocity of the electromagnetic wave is the approximate speed of light in a vacuum, so the propagation time differences from different nodes are quite minor and can be ignored in a subspace. The synchronization protocol based on RBS suffers from the uncertainties of the transmission and reception times, as well as the yield synchronization error, which is about 13 μs [30], and cannot meet the requirements of the UIPS. Several modifications are performed to reduce the error. First of all, the synchronization and other data transmissions have the same communication module as in the other system, but there are two different modules in the UIPS, and the modules will not be occupied by the data transmission process. The interrupt for transmitting and receiving have the highest priority, and will not be terminated by the other interrupt.

**Figure 6:**
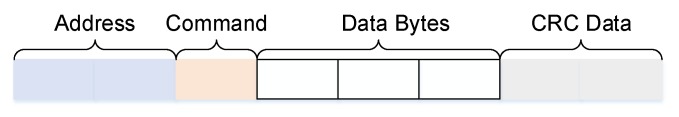
The structure for the data frame, CRC: cyclic redundancy check.

As described above, the rest factors for synchronization accuracy are in the RF module. CC2500 is selected as a synchronization module in UIPS, which can provide extensive hardware support for packet handling, data buffering, and burst transmissions [31]. Encoding/decoding, transmitting/receiving, and alignment are affected by the traffic rate of the synchronization module. A testing experiment is designed in order to verify this and calculate accuracy. The reference node transmits eight bytes of synchronization data to the other node. The structure for the data frame is shown in Figure 6.

The length of the address is two bytes in the figure, and a command is used to distinguish data frames into time synchronization or data transmission. This takes up one byte. The next three bytes are data bits. Because data transmission is completed by WiFi module, the data bits are reserved for future research. The last two bytes are cyclic redundancy check (CRC) bits, which indicates whether the received data has some errors. There are two nodes in the experiment, the data frames are sent once every 200 ms, the receiver will return the data to the reference immediately when the receiving is finished. The time from the beginning of transmission to the end of receiving for the reference node is recorded as $t_{s}$ in the sender. The statistics of 500 measurements for every rate are shown in Table 1.

The difference between the maximum and minimum value of $t_{s}$ can indicate the precision of the time synchronization. This brings us to the conclusion that the higher the data communication rate is, the lower the differences are. The difference will be 1 μs when the rate is above 100 kb/s, so the precision of the time synchronization should be less than 1 μs because of one-sided communications in UIPS. The ranging measurement error, resulting from this, is less than 0.34 mm, assuming the ultrasonic velocity is 340 m/s.

### 3.2. Ultrasonic TOF Estimation

The analog-to-digital converter (ADC) of the received nodes starts to sample the ultrasound signal using a sampling frequency $f_{s}$ ($f_{s}=\frac{1}{T}$) when the synchronization data are arriving. The range of ultrasonic propagation is limited to 5 m in UIPS, so the time it takes to sample the signal is less than 15 ms, assuming the velocity is 340 m/s. The uncertain drift of the crystal oscillator of the received nodes can be ignored in this period. Therefore, the time instant at which the envelope reaches its maximum values ($n_{max}$) can be calculated with $\frac{n_{max}}{f_{s}}$.

The parabolic interpolation method in which the adjacent samples around $\hat{A}\left(n_{max}\right)$ can reduce the TOF estimation ($t_{tof}$) error [32], is given by:

(16) $$t_{tof}=\frac{n_{max}+n_{x}}{f_{s}}$$

where $n_{x}$ is the fractional correction term obtained from parabolic interpolation with three samples [27], given by:

(17) $$n_{x}=\frac{\hat{A}\left(n_{max}-1\right)-\hat{A}\left(n_{max}+1\right)}{2\cdot \left[\hat{A}\left(n_{max}-1\right)-2\hat{A}\left(n_{max}\right)+\hat{A}\left(n_{max}+1\right)\right]}$$

The phase information at $n_{x}$ can be obtained from Equation (15), and it can improve a greater resolution of $t_{tof}$. The TOF calculated value by phase is shown in Equation (19) [20], where Int[] is the integer operation.

(18) $$t_{tof}=Int\left[\frac{n_{max}+n_{x}}{f_{s}}\right]+\frac{\hat{\theta }\left(n_{max}\right)}{2\pi f_{s}}$$

### 3.3. Estimation of Distances and Positioning

The speed of ultrasound *v* in air depends on many factors. The influence of temperature is only considered in the special indoor condition, and it can be estimated with Equation (19) [33], where ϕ is the temperature in degrees Celsius,

(19) v=(331.3+0.6ϕ)m/s

Then, the distance $d_{i}$ between the target node and the anchor node *i* can be estimated with $t_{tof}$ and *v* as:

(20) $$d_{i}=v\cdot t_{tof}$$

The coordinate of the anchor node *i* is $\left(x_{i},y_{i},z_{i}\right)$, and it is assumed that the number of the anchor nodes is *k*. The coordinate of the target node is (x,y,z). Then, these equations are obtained as:

(21) $$\begin{array}{cc} {\left(x-x_{1}\right)}^{2}+{\left(y-y_{1}\right)}^{2}+{\left(z-z_{1}\right)}^{2} & =d_{1}^{2}\\ {\left(x-x_{2}\right)}^{2}+{\left(y-y_{2}\right)}^{2}+{\left(z-z_{2}\right)}^{2} & =d_{2}^{2}\\ {\left(x-x_{3}\right)}^{2}+{\left(y-y_{3}\right)}^{2}+{\left(z-z_{3}\right)}^{2} & =d_{3}^{2}\\ \cdots \;\;\;\;\;+\;\;\;\;\;\;\cdots \;\;\;\;\;+\;\;\;\;\;\;\cdots \;\;\; & =\cdots \\ {\left(x-x_{k}\right)}^{2}+{\left(y-y_{k}\right)}^{2}+{\left(z-z_{k}\right)}^{2} & =d_{k}^{2} \end{array}$$

where the left and right equations are subtracted from the first and second equations, respectively, and the equation is expressed in matrix form as:

(22) A·x=b

where A, x and b are referred to as:

(23) $$A=\left[\begin{array}{ccc} 2(x_{1}-x_{2}) & 2(y_{1}-y_{2}) & 2(z_{1}-z_{2})\\ 2(x_{1}-x_{3}) & 2(y_{1}-y_{3}) & 2(z_{1}-z_{3})\\ 2(x_{1}-x_{4}) & 2(y_{1}-y_{4}) & 2(z_{1}-z_{4})\\ \vdots & \vdots & \vdots \\ 2(x_{1}-x_{k}) & 2(y_{1}-y_{k}) & 2(z_{1}-z_{k}) \end{array}\right]$$

(24) $$x={\left[\begin{array}{ccc} x & y & z \end{array}\right]}^{T}$$

(25) $$b=\left[\begin{array}{c} x_{1}^{2}-x_{2}^{2}+y_{1}^{2}-y_{2}^{2}+z_{1}^{2}-z_{2}^{2}+d_{2}^{2}-d_{1}^{2}\\ x_{1}^{2}-x_{3}^{2}+y_{1}^{2}-y_{3}^{2}+z_{1}^{2}-z_{3}^{2}+d_{3}^{2}-d_{1}^{2}\\ x_{1}^{2}-x_{4}^{2}+y_{1}^{2}-y_{4}^{2}+z_{1}^{2}-z_{4}^{2}+d_{4}^{2}-d_{1}^{2}\\ \vdots \\ x_{1}^{2}-x_{k}^{2}+y_{1}^{2}-y_{k}^{2}+z_{1}^{2}-z_{k}^{2}+d_{k}^{2}-d_{1}^{2} \end{array}\right]$$

The close solution of Equation (22) for x can be obtained by using the least squares method from Equation (26) [27].

(26) $$x={\left(A^{T}\cdot A\right)}^{-1}\cdot \left(A^{T}\cdot \vec{b}\right)$$

## 4. Implementation of the UIPS

The implementation of UIPS is based on embedded system design. A 32-bit ARM Cortex™-M7 microprocessor is selected as the hardware, which features a single floating point unit (SFPU) and supports single precision data processing instructions and data types. The software is programmed in a bare metal machine in which the registers of hardware can be read or written directly. The real-time performance of the system is strengthened significantly.

### 4.1. Hardware

**Figure 7:**
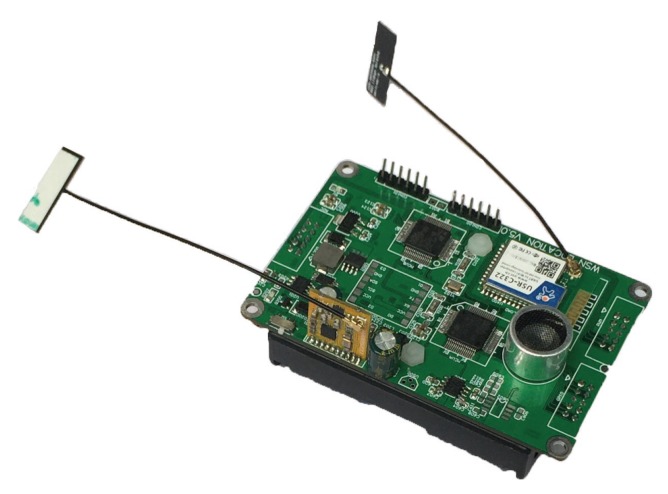
The photo of the UIPS node.

The node hardware of UIPS is designed for the core board with a size of 90 mm × 58 mm, shown in Figure 7. The core board is powered by a lithium battery (LB), which can be charged for reuse. It can be configured as a receiver anchor node and arranged in the interior ceiling. The specified extended board for the core board that is configured as the transmitter node can be used to control the mobile robot.

**Figure 8:**
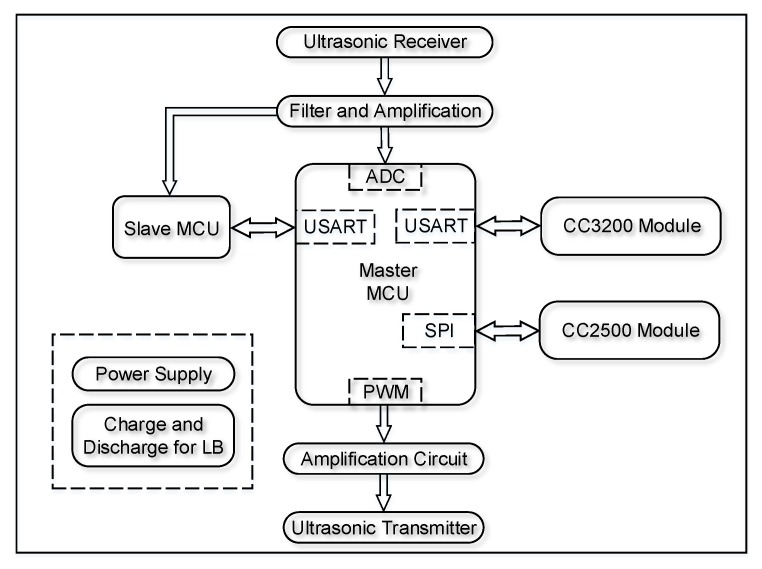
The hardware architecture of the UIPS node. ADC: analog-to-digital converter; LB: lithium battery; MCU: microcontroller unit; PWM: pulse width modulation; SPI: serial peripheral interface.

The microcontroller unit (MCU), the transmitter and receiver for ultrasound, the CC3200 and CC2500 modules, and the power system comprise the hardware system of UIPS, which is shown in Figure 8. UIPS has two STM32F722RCT6 32-bit MCUs, which have a running frequency up to 216 MHz and come from STMicroelectronics (http://www.st.com); one is the master, and the other is the slave with alternate sampling and calculation. This MCU integrates 512 Kbytes Flash memory with protection mechanisms and 256 Kbytes of static random access memory (SRAM) on chip. It has an abundance of peripheral resources and interfaces, such as a 12-bit ADC, a digital to analog converter (DAC), a timer that can produce the pulse width modulation (PWM) signal, universal synchronous or asynchronous receiver transmitters (USARTs) and a direct memory access (DMA) channel [34].

The signal strength of the ultrasonic transmitted transducer with a frequency of 40 kHz is too low to be sampled by the ADC directly, so it must be amplified firstly, and then processed by a band-pass filter to reduce the out-of-band noise. The ultrasonic received sensor can be driven by the PWM orsinusoidal signal, which can be generated by DAC. The former is adopted in UIPS. It can be obtained from the timer with high precision.

The CC3200 [35] module communicates with the master MCU by USART, which can realize the transparent data transmission between the master and the server via the WiFi wireless network. The CC2500 module is used for time synchronization and communicates with the master MCU through the SPI interface. Besides, the module is linked to the master MCU via an input/output (IO) port in hardware.

### 4.2. Software

The implementation of the algorithm for envelope detection in Section 2.2 requires a large number of matrix calculations using Equation (13), but some regularity can be found if Equation (10) is plugged into Equation (13). The elements of $\Psi_{i}$ will be periodic and constant if the length *N* and sampling period *T* are constant, so all ${\left(\Psi_{i}^{T}\Psi_{i}\right)}^{-1}\Psi_{i}^{T}$ will also form a series of periodic coefficients. The coefficients in only one period are enough for the implementation of the algorithm, and are computed in advance using MATLAB and are written into the read-only memory (ROM) of the MCU in a table form. The computational complexity of the algorithm can be reduced significantly.

There are rich DMA channels and interrupt vectors on the MCU chip, with which the real-time and reliability performance of the software system can be enhanced. There is a joined IO port between the CC2500 module and the master MCU as described above. The module will produce a rising edge pulse signal when the time synchronization data come, which makes the software of the master go into an external interrupt vector with the highest priority of all. The system will have an alternating sampling and detection between the master MCU itself and the slave MCU, shown in Figure 6. The master will turn on the DMA channel and enable the timer to trigger the ADC for ultrasound signal acquisition with rate $f_{s}$ if it is time for the master itself. The software will run into the DMA interrupt service program when all *M* pieces of data are collected.

**Figure 9:**
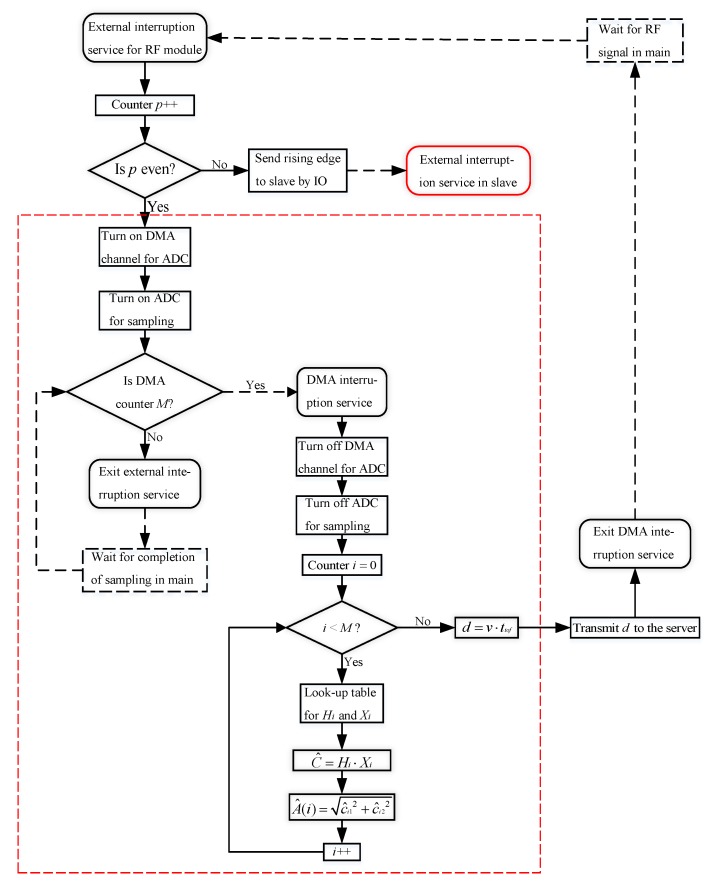
The software architecture and flow diagram of the UIPS receive node. DMA: direct memory access. IO: input/output.

The envelope detection method presented in Section 2.2 is implemented in the DMA interrupt service program and then calculates the distance value from the target node to the anchor node; $H_{i}={\left(\Psi_{i}^{T}\Psi_{i}\right)}^{-1}\Psi_{i}^{T}$ in the figure. The value will be transferred to the server through the WiFi network. The slave MCU will obtain the distance value like the master in the external interrupt program if it is its time, shown in the red dashed rectangle of the figure, and then transfer the value to the master through USART; finally, the master transfers it to the server. The whole software flow diagram is shown in Figure 9.

## 5. Experiments

A simulation experiment using MATLAB is described to prove the validity of the envelope detection method in this section, meanwhile, the point-to-point ranging and the position for the mobile robot are presented to validate the UIPS’s performance.

### 5.1. Simulation

**Figure 10:**
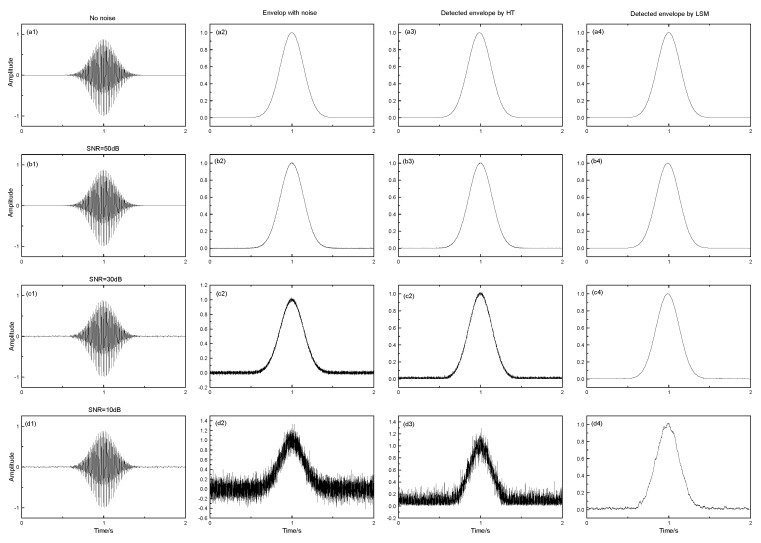
The comparison for envelope detection based on the Hilbert transform (HT) and least squares method (LSM).

The analog ultrasonic signal and its envelope are generated from Equations (1) and (2) in Section 2.2, which only has a peak, as mentioned earlier. Noise is inevitable in any practicable system, so the white Gaussian series are added to the signals with a different signal-to-noise ratio (SNR) in order to test the filtering effect of the algorithm in Section 2.2. The Hilbert transform (HT) is widely used for envelope detection in engineering [36], by which the ultrasonic envelope is also extracted for comparison with the method in this paper. The results are shown in Figure 10.

The envelope detection method based on LSM can detect the envelope of ultrasound with a high precision, comparing (a2) and (a4) in the figure, and there is no time delay for the peak between them. Filter effect analysis proves that the proposed algorithm can obtain a good filtering result, even if the SNR is very low (as shown from (b) to (d) of Figure 10). The method based on HT can detect the envelope too, but it has no filter effect from the third column on. This can only be achieved by means of some special digit filter. Besides, the HT is more complex.

### 5.2. Applications

The distance measurement experiment is completed to evaluate the accuracy of UIPS. A pair of nodes are at the same level as each other, which can guarantee that the nodes work on the LOS condition, avoiding interference for ultrasonic multi-path propagation. One is configured as the transmitter, and the other node as a receiver. The transmitter sent a PWM of 1 ms under the control of the time synchronization signal with a period of 200 ms. The ultrasonic signal completely disappears in such time, so that the influence of the last cycle will be avoided.

**Figure 11:**
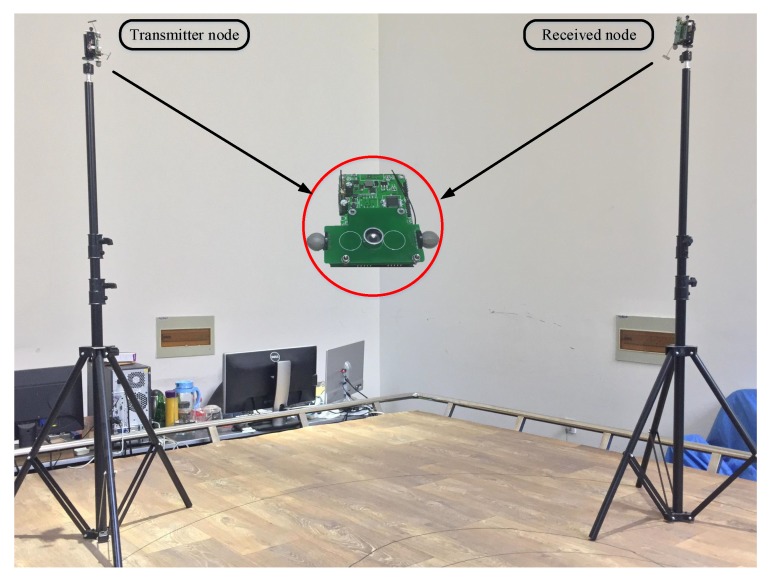
An illustration of the distance measurement between two nodes.

A commercial vision-based localization system (vicon) from Oxford Metrics Limited (www.vicon.com) is used to measure the separation between nodes, which has a precision of 1 mm. Firstly, some optics markers are planted around the ultrasound transducer symmetrically, shown in Figure 11, so that the position coordinates of two nodes can be obtained from the vicon system. They are $\left(x_{1},y_{1},z_{1}\right)$ and $\left(x_{2},y_{2},z_{2}\right)$, and the distance is:

(27) $$d=\sqrt{{\left(x_{1}-x_{2}\right)}^{2}+{\left(y_{1}-y_{2}\right)}^{2}+{\left(z_{1}-z_{2}\right)}^{2}}$$

Eight group distances are measured, which have some random bits. The quadrature sampling (QS) [27] is achieved by UIPS to compare the method based on LSM in this paper. The statistics are shown in Table 2, where $\bar{X}$ is an average of 500 times of measurement for each distance, var is their variance, and ${\left|E\right|}_{max}$ is defined as:

(28) $${\left|E\right|}_{max}=\frac{1}{2}\left(v_{max}-v_{min}\right)$$

where $v_{max}$ and $v_{min}$ represent respectively the maximum and minimum in all 500 measurements. ${\left|E\right|}_{max}$ can reflect a certain accuracy of UIPS.

We can see that $\bar{X}$ error is less than 1.5 mm with the reference distance for two methods. The highest ${\left|E\right|}_{max}$ and var can obtain values of 0.61 and 0.23, but they will increase with distance. This is because of the influence of noise when the ultrasonic signal becomes weakened, and the SNR is smaller. ${\left|E\right|}_{max}$ and var in LSM are smaller than those in QS in all groups, so the method based on LSM has a higher accuracy and robustness.

**Figure 12:**
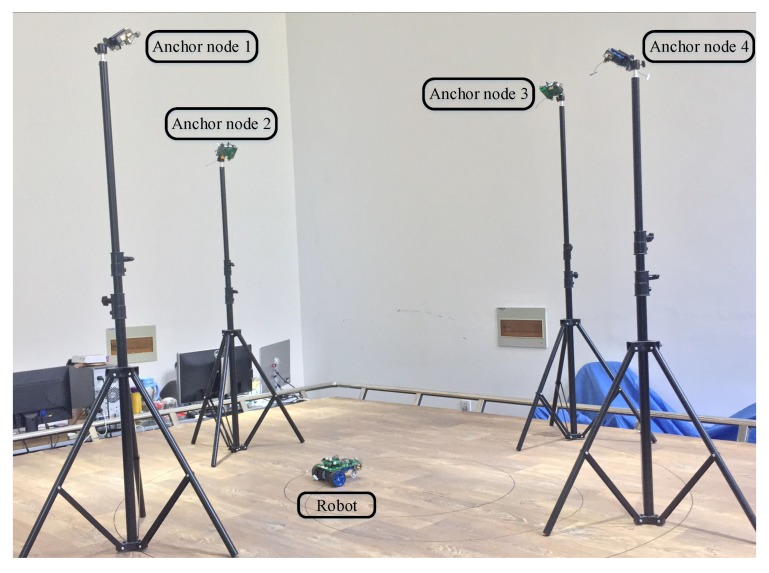
An illustration of the positioning experiment for the mobile robot.

The localization experiment for the mobile robot is performed in order to test the system function. Four anchor nodes are placed at a fixed position, which are configured as the receiving end, shown in Figure 12. Here, the coordinates of the anchor node are obtained from vicon as above. They are, respectively: (−115.79,1004.64,1501.64), (−1024.88,−44.74,1685.52), (18.60,−966.18,1577.05), and (968.89,−44.40,1914.52). Each transducer of the anchor node has common coverage areas where the signal from the robot can be received by all. A and b can be calculated with Equations (23) and (25), given by

(29) $$A=\left[\begin{array}{ccc} 1818.18 & 2098.76 & -367.76\\ -268.78 & 3941.64 & -150.82\\ -2169.36 & 2098.08 & -825.76 \end{array}\right]$$

(30) $$b={\left[\begin{array}{ccc} -615.73+{d_{2}}^{2}-{d_{1}}^{2} & -143.30+{d_{3}}^{2}-{d_{1}}^{2} & -1328.47+{d_{4}}^{2}-{d_{1}}^{2} \end{array}\right]}^{T}$$

The motion with open-loop trajectory for the robot is a standard circle, which starts from different positions with various radii. There are also some optics markers around the ultrasound transducer of the node on the robot, so the trajectory can be obtained from vicon. The mobile robot will transmit the ultrasonic signal in each 200 ms cycle as with the distance measurement. The signal is transmitted with LOS in order to overcome the multi-path case, additionally, a short ultrasonic signal pulse, just 1 ms in this paper, can reduce interference for ultrasonic multi-path effect [37], so it is ignored here. The robot moves with a very low speed in the common coverage areas, so the Doppler effect of ultrasound is ignored in this experiment.

**Figure 13:**
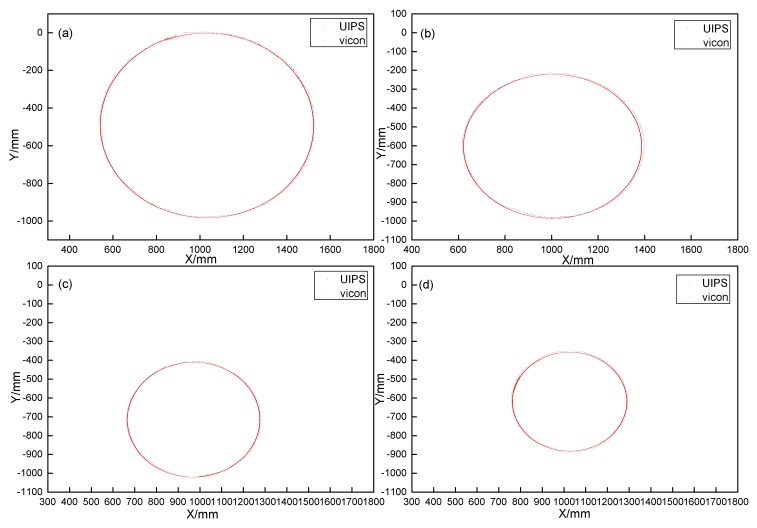
The comparison of the robot motion with different radii circular trajectories from (**a**–**d**). The position data sampling rate from vicon is much greater than that of UIPS.

Four experiments are conducted with different radii in total, and the trajectory tracking results are shown in Figure 13.

We can see that the UIPS can follow the tracks of the robot with a maximum error of 10.24 mm. The error is 12 mm when the same experiments are completed though quadrature demodulation [38] with the same sampling frequency $f_{s}$. ${\left|E\right|}_{m}$ is used to represent the maximum error in this paper, and is defined as:

(31) $${\left|E\right|}_{m}=\sqrt{{\left(x_{m}-x_{r}\right)}^{2}+{\left(y_{m}-y_{r}\right)}^{2}+{\left(z_{m}-z_{r}\right)}^{2}}$$

where $\left(x_{m},y_{m},z_{m}\right)$ is a position from UIPS, which deviates furthest from the vicon reference. $\left(x_{r},y_{r},z_{r}\right)$ is from vicon, because vicon is not in time synchronization with UIPS and has a much higher sampling frequency for positioning data than that of UIPS. $\left(x_{r},y_{r},z_{r}\right)$ is the nearest point to $\left(x_{m},y_{m},z_{m}\right)$ in all cases.

## 6. Conclusions

In this work, we present a new envelope detection method for the ultrasonic signal and develop an indoor position system based on this method, which can be also applied to other fields. Filter effect analysis from simulation proves that the method is effective. The system can fix a continuous position on the mobile robot with high and robust stability. We also present an improved time synchronization method based on RBS in the UIPS, which has an accuracy up to 1 μs. Network nodes have individual communication modules using the WiFi protocol, which is used widely in day-to-day life. Therefore, we will integrate the ultrasonic transducer with a mobile phone in the future and realize the precise positioning for personnel using the phone and the UIPS.

## Figures and Tables

**Table 1:** The statistics of the time synchronization experiment with different communication rates.

Data Communication Rate	5 kb/s	10 kb/s	20 kb/s	50 kb/s	100 kb/s	150 kb/s
Maximum of $t_{s}$ (μs)	33,200	17,059	8986	4242	2528	1991
Minimum of $t_{s}$ (μs)	33,185	17,052	8982	4140	2527	1990
Difference (μs)	15	7	4	2	1	1

**Table 2:** The statistics of distance measurements. QS: quadrature sampling.

Ref (mm)		1195.16	1454.86	1666.49	1977.27	2277.89	2605.79	2877.75	3150.92
$\bar{X}$	LSM	1195.83	1453.77	1667.61	1977.58	2278.46	2606.49	2878.31	3151.36
	QS	1194.34	1453.04	1666.91	1976.38	2277.25	2506.37	2878.09	3150.61
${\left|E\right|}_{max}$	LSM	0.61	0.68	0.75	0.96	1.14	1.21	1.33	1.42
	QS	1.34	1.67	1.69	2.30	1.90	1.82	3.06	2.84
var	LSM	0.23	0.26	0.27	0.34	0.21	0.22	0.37	0.40
	QS	0.41	0.60	0.38	0.70	0.76	1.02	1.14	1.13
